# Long-Term Management of Patients with Mild Urea Cycle Disorders Identified through the Newborn Screening: An Expert Opinion for Clinical Practice

**DOI:** 10.3390/nu16010013

**Published:** 2023-12-20

**Authors:** Albero Burlina, Serena Gasperini, Giancarlo la Marca, Andrea Pession, Barbara Siri, Marco Spada, Margherita Ruoppolo, Albina Tummolo

**Affiliations:** 1 Division of Inherited Metabolic Diseases, Reference Centre for Expanded Newborn Screening, University Hospital of Padova, 35128 Padova, Italy; 2 Inherited Metabolic Unit Disorders, Pediatric Department, Fondazione IRCCS San Gerardo dei Tintori, 20900 Monza, Italy; serena.gasperini@irccs-sangerardo.it; 3 Newborn Screening Lab, IRCCS Meyer Children’s Hospital, Department of Experimental and Clinical Biomedical Sciences, University of Florence, 50139 Firenze, Italy; giancarlo.lamarca@meyer.it; 4 Pediatric Unit, IRCCS Azienda Ospedaliero-Universitaria di Bologna, 40138 Bologna, Italy; andrea.pession@unibo.it; 5 Division of Metabolic Diseases and Hepatology, Bambino Gesù Children’s Hospital, IRCCS, 00165 Rome, Italy; barbara.siri@opbg.net; 6 Department of Pediatrics, University of Turin, 10124 Turin, Italy; marco.spada@unito.it; 7 Department of Molecular Medicine and Medical Biotechnology, University of Naples, Federico II, 80138 Naples, Italy; margherita.ruoppolo@unina.it; 8 CEINGE–Biotecnologie Avanzate S.C.A.R.L., 80145 Naples, Italy; 9 Department of Metabolic Diseases and Clinical Genetics and Diabetology, Giovanni XXIII Children Hospital, Azienda Ospedaliero-Universitaria Consorziale, 70126 Bari, Italy; albina.tummolo@policlinico.ba.it

**Keywords:** urea cycle disorders, expanded newborn screening, nitrogen scavengers, follow-up

## Abstract

Urea cycle disorders (UCDs) are a group of rare inborn errors of metabolism caused by a deficiency in one of the six enzymes or one of the two transporters involved in the urea cycle. Current guidelines suggest that early diagnosis and treatment of mild UCDs may improve survival and prevent decompensation and neurocognitive impairment. Nevertheless, clinical studies are very difficult to carry out in this setting due to the rarity of the diseases, and high-level evidence is scant and insufficient to draw conclusions and provide clinical guidelines. With the early introduction of newborn screening, the Italian healthcare organization fostered an advancement in expertise in metabolic disease management and screening programs, by allocating resources, and favoring the expansion of newborn screening. A group of experts operating in Italian centers decided to share their experience and provide advice for the management of mild UCDs in clinical practice. A consensus was reached by the Estimate–Talk–Estimate (ETE) method. Five items were identified, and statements for each item were agreed. Briefly, the panel advised completing the diagnosis by expanded newborn screening (ENS) with biochemical and genetic confirmation and by following up with the patient during the first year of life, with a routine laboratory and metabolic profile as well as with clinical observation. Early initiation of therapy is advised and should be followed by therapy adjustment once the diagnostic profile is completed. The therapy should be based on a low-protein diet and nitrogen scavengers. The long-term follow-up is based on growth and nutritional assessment, clinical and neurocognitive evaluation, and laboratory and instrumental parameter monitoring.

## 1. Introduction

**Figure 1:**
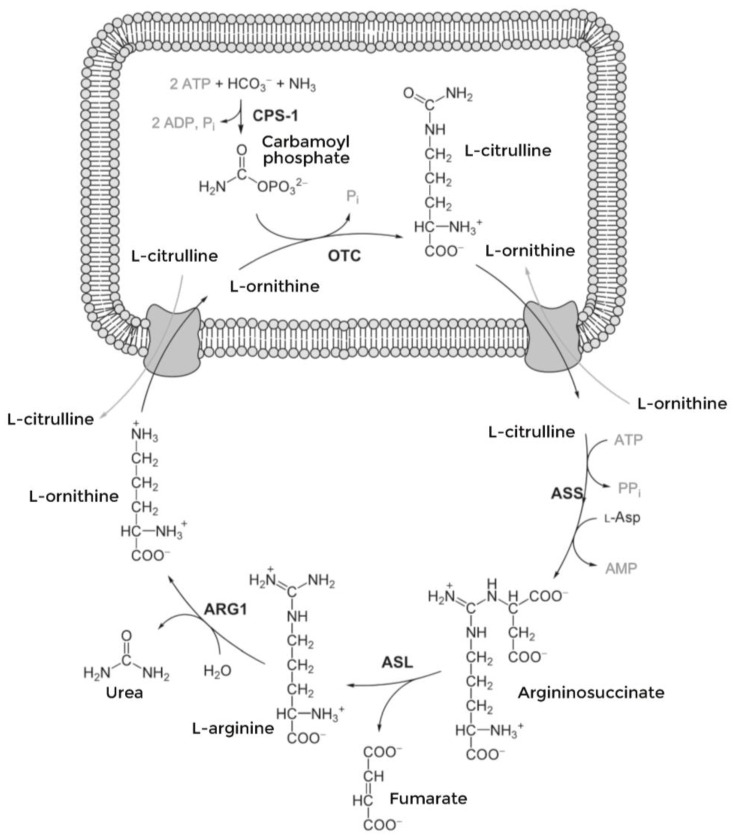
Urea cycle.

Urea cycle disorders (UCDs) are a group of rare inborn diseases caused by either a deficiency in one of the six enzymes—carbamoylphosphate synthetase I (CPS1), ornithine transcarbamylase (OTC), argininosuccinic acid synthetase (ASS1), argininosuccinic acid lyase (ASL), arginase (ARG1), *N*-acetyl glutamate synthetase (NAGS)—or one of the two transporters—ornithine translocase (ORNT1) and citrin (aspartate/glutamate carrier)—involved in the urea cycle (Figure 1) [1].

UCDs include distal-type diseases, i.e., citrullinemia type I, argininosuccinic aciduria, and argininemia, mitochondrial UCDs (*CPS1*, *OTC* and *NAGS1*), which are not currently included in the Italian ENS panel, and secondary conditions such as citrin deficiency and hyperornithinemia–hyperammonemia–homocitrullinuria (HHH) syndrome [2]. Epidemiological studies on UCDs is challenging as routine expanded newborn screening (ENS) is not universally applied, and a lack of disease awareness may prevent early diagnosis or lead to misdiagnosis [3]. Cumulative incidences of UCDs were reported between 1:35,000 and 1:69,000, with ornithine transcarbamylase deficiency having an incidence of 1:69,904 live births in Italy in the late 1990s [4].

Newborn screening (NBS) is a clinical and laboratory practice that identifies, shortly after birth, pre-symptomatic conditions that can affect a child’s long-term health and survival. NBS was introduced in Europe in the 1960s with the screening for phenylketonuria; the panel of screened conditions gradually increased due to the introduction of tandem mass spectrometry (MS/MS), introducing the practice of ENS. This technique allowed newborn children to be screened before discharge for several conditions using a single dried blood spot [5].

Severe or total absence of enzyme activity is associated with complete urea cycle disruption, ammonia accumulation and clinical presentation in the newborn period. Mild or partial enzyme deficiencies may manifest at any stage of life, leading to chronic symptoms. Triggers such as starvation and/or illnesses may result in organ damage caused by ammonia accumulation. Clinical symptoms of UCDs include loss of appetite, vomiting, lethargy, and behavioral abnormalities, with growth deficiency and neurological impairment worsening over time [6,7]. Current guidelines state that early diagnosis and treatment of mild UCDs may improve survival and prevent decompensation and neurocognitive impairment [6]. Nevertheless, clinical studies are very difficult to carry out in this setting due to the rarity of these diseases, and high-level evidence is scant and insufficient to draw conclusions and provide clinical guidelines. In 2019, Häberle published ‘proposed guidelines’ providing advice for clinicians [6]. However, the proposed guidelines were unable to offer definite and specific recommendations for the long-term management of mild or late-onset UCDs [6]. Therefore, the need for an early diagnosis, criteria for patients’ treatment and long-term follow-up in mild or late-onset of UCDs remain unmet.

Italy pioneered the introduction of NBS for metabolic diseases, which included distal UCDs [2]. Ever since 2016, this standard practice by the Italian national health system promoted the expertise in metabolic disease management and screening programs establishing multiple centers of excellence. A group of experts operating in such centers (with approximately 70 cases of distal UCDs diagnosed by ENS) decided to share their experience and provide advice for the management of mild UCDs in clinical practice, given the need for guidelines tackling these rare conditions. Due to the limited number of experts involved in the project, a consensus was reached by the Estimate–Talk–Estimate (ETE) method, or ‘mini-Delphi’ [8,9].

This article reports the authors’ advice on some pressing issues, including the role of ENS in diagnosing distal UCDs, the correlation of genotype with the disease phenotype, therapy and follow-up. It is addressed to centers that manage patients diagnosed with mild UCDs either by ENS, when possible, or by other diagnostic means.

## 2. Methods

### 2.1. Panel Selection

**Figure 2:**
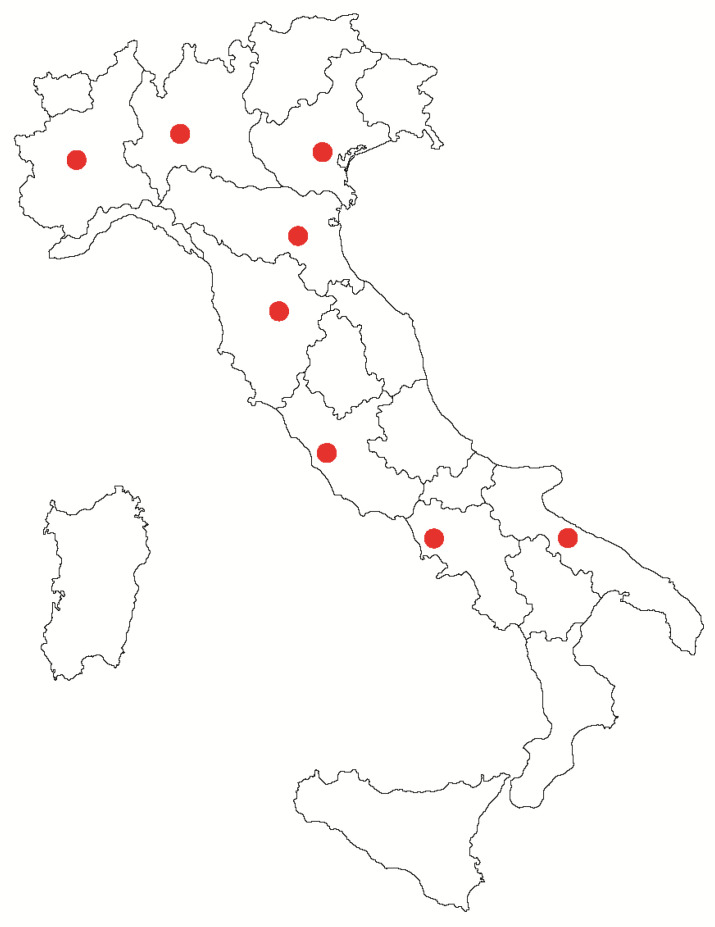
Participating center localization in Italy. Red dots represent the location of centers.

Authors of this article include six metabolic pediatricians from the most prominent Italian pediatric, metabolic and rare diseases centers, with expertise in managing and treating UCDs patients, as well as two biochemists experts in biochemical and genetic confirmation of ENS-positive results (Figure 2).

### 2.2. Design

**Figure 3:**
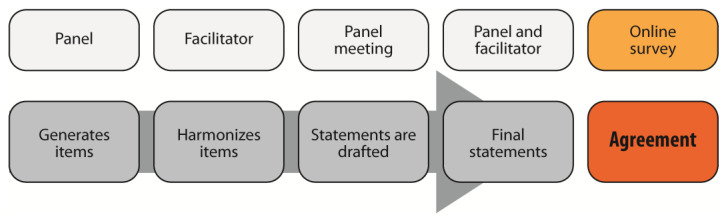
Workflow of the Estimate–Talk–Estimate process.

The ETE is a method for reaching a consensus within a selected group of experts. It combines a nominal group activity restricting verbal interaction with face-to-face interaction processes. Figure 3 shows the project workflow. The panel sought the assistance of a facilitator (Polistudium srl, Milan, Italy) whose role includes material preparation, meeting facilitation, and methodological accuracy.

Firstly, experts individually generated defined points of interest (item) that deserved to be explored and discussed according to their experience and the available evidence. The facilitator sift through these items, which were then presented to the expert group. During the meeting, finalized items were used by clinicians and laboratory specialists to draft statements for each item. The first session of the meeting resulted in a certain number of statements. In the second and last session of the face-to-face meeting, the experts and the facilitator reviewed and further discussed the statements, reaching a final version. A consensus among the panel experts on the generated statements was expressed through an online platform (SurveyMonkey, San Mateo, CA, USA) after the meeting. Participants anonymously rated their level of agreement or disagreement using a Likert scale of 1–5, with 1 indicating complete disagreement and 5 indicating complete agreement. An agreement was reached when at least 75% of responders voted 4 or 5 [10].

## 3. Results

In the described process, the panel identified five items—the role of ENS in distal UCDs diagnosis, the correlation of genotype and phenotype for diagnosis confirmation, diet therapy, pharmacological therapy, and follow-up. Twenty-three final statements were produced. Agreement was reached on all statements, and the level of agreement was 100% in 18 cases and 87% in five cases. The final items, statements and the degree of consensus for each of them are reported in Table 1.

### 3.1. The Role of Expanded Newborn Screening in Distal UCDs 

Since 2016, ENS using MS in Italy currently includes the screening of distal UCDs [2]. More UCDs could be included in the screening panel once a reliable diagnostic test is available, which is a key requirement for inclusion [11]. Possible markers for OTC and CPS 1 deficiency are low blood levels of citrulline (Cit) or high levels of glutamine (Gln). Unfortunately, many protein-restricted and preterm newborns also have low blood Cit [12], while Gln is unstable and is destroyed during MS/MS analysis [13]. Additionally, the assessment of orotic acid is a sensitive but low specificity test according to the authors’ experience, while blood hypocitrullinemia is not a reliable marker for OTC deficiency with MS/MS-based NBS [14]. Therefore, proximal urea cycle enzyme defects are not yet efficiently detected [15].

Overall, two leading counsels are provided for early diagnosis of mild UCDs. Firstly, the panel advises that a patient with a positive result for distal UCDs by ENS is initiated for treatment without delay. In parallel, the diagnostic process should be continued throughout the first year of life, considering clinical and metabolic parameters and family history. This prolonged follow-up can detect the relevance of the molecular diagnosis and direct long-term management. Secondly, the authors agreed during the discussion that high citrulline levels are a reliable risk marker. Nevertheless, a few participants disagreed on the statement “If the citrulline levels exceed 100 μmol/L, it is crucial to recall the patient immediately” as this result should be connected to the difficulty of establishing 100 μmol/L as a robust high cut-off level. The value of 100 μmol/L is based on the literature [16], but the panelists warn that laboratory methods are not yet universally standardized, and different cut-off values could possibly be envisaged depending on the methodology used. 

### 3.2. Biochemical Assessment

The statement “Under an emergency regime, a biochemical confirmation test based on plasma amino acid assessment is mandatory for the diagnosis and specific treatment” suggests that a newborn with suspected distal UCDs must receive a prompt laboratory testing to direct the therapy as patients identified with a distal UCDs must have early management. The differential diagnosis of distal UCDs can be obtained in an early phase by amino acid assessment (citrulline and arginin succinic acid) in the blood and orotic acid in the urine. Mild and very mild forms of citrullinemia type I can be distinguished based on citrulline serum levels. Those with levels above 100 μmol/L should be considered mild, while those with levels between 40 and 100 μmol/L should be classified as very mild. Interpreting mildly elevated citrulline is still challenging, and several conditions, as well as heterozygous state for citrullinemia type 1, must be included in the differential diagnosis [17].

Genetic testing has replaced enzyme activity measurement as definitive confirmatory test. Patients identified as affected with distal UCDs by ENS must have early management, independently of the timing to receive genetic confirmation [6].

### 3.3. Role of Genetic Testing

The genetic assessment may use single-gene testing if the biochemical findings indicate that mutation of a particular gene is most likely. A multigene panel that includes several genes linked to urea cycle defects [*ASS1*, *ASL*, *ARG1*, *CPS1*, *DLD*, *NAGS*, *OTC*, *PC*, *SLC25A13 (citrin deficit)*, *SLC25A15* (*HHH*), *SLC7A7* (*LPI*)] may be considered when clues to the gene mutation are missing. A second-line genetic screening is advised if no mutations are found in the genes involved in the ENS panel. 

Genetic testing is necessary for a complete diagnosis and further interventions, but the management of patients must start even before the testing is completed. Alas, the clinical relevance of the genetic diagnosis is inconsistent among all cases [17]. Certain mutations clearly link to specific clinical manifestations but suffer from incomplete penetrance [18,19,20]. The clinical interpretation of genetic variants of uncertain significance (VUS) needs further information about the condition’s natural history. Even patients carrying homozygous devastating mutations (such as nonsense, splicing, large deletions, or translation initiation codon mutations) may develop the disease later in time [19].

The final statement of this section, “Prediction of enzymatic defect for severe form, assessed by biallelic expression system and positive family history, should guide the pharmacological treatment in the pre-symptomatic period”, found a 100% agreement. The authors are in concordance for advising an immediate treatment in asymptomatic patients with a positive ENS result and a positive family history to prevent the acute events of severe distal UCDs.

### 3.4. Treatment and Follow-Up

Due to the difficulty in generating high-level evidence-based guidelines, and in detecting the severity of the condition, even when enzyme-based tests are performed [21], the group of experts produced a protocol for treatment and follow-up based on their clinical practice. They are aware, nevertheless, that the impact of ENS on outcomes may be very variable and that counsels must be tailored to the local management capabilities.

The authors advise not to adopt a wait-and-watch approach for newborns with a positive result at ENS due to the severity of possible acute events. Preventative intervention is advised with dietary and pharmacologic treatments since the molecular diagnosis. The follow-up in the first year of life, with enzyme-based test and clinical observation, complete the diagnosis. The therapeutic strategy may be personalized or customized to each patient’s condition after the first year.

Authors agree that diet therapy is a mainstay of all UCDs management based on physiopathology criteria and broad clinical experience [6]. When a distal UCDs is diagnosed by ENS, a low-protein diet must be proposed to allow growth and prevent endogenous catabolism while ensuring the necessary protein requirements. Adequate protein and energy supply can be based on the FAO/WHO/UNU 2007 “safe levels of protein intake”, with a maximum of 20–30% of the total protein intake provided as essential amino acids [6,22].

Nutritional counseling must be offered early. In the first year of life, regular dietetic evaluation, growth assessment, and weaning instruction should be provided to the family [23]. In the following years, during catabolic events (gastroenteritis, fever, starvation) or protein overload, patients should be treated with an integrated approach of aetiologic and supportive therapy and a low-protein diet with arginine supplementation [24]. Acute events in patients with UCDs are treated equally regardless of the severity [6]. Indeed, catabolic events are always linked to the risk of decompensation and should always be considered as emergency events, independently of their frequency. 

The statement “When dealing with mild UCDs, especially in late-onset forms diagnosed through NBS, the switch from a ‘wait and see’ approach to an active therapeutic approach should be based on the abnormality of biochemical data and/or genotype profile” was supported by two main considerations. 

Although diet is safe and inexpensive, starting a diet treatment after a free diet regimen for the first follow-up period can represent an additional challenge for both the patient and the family, resulting in inadequate compliance and/or arbitrary discontinuation of diet [25]. Breastfeeding should always be encouraged regardless of the type of approach implemented.

In asymptomatic patients, plasma ammonia and amino acids can be assessed at 24 h of age; otherwise, follow the current UCDs guidelines in case of symptoms [6]. These principles are also applied to proximal UCDs (OTC, CPS1, and NAGS) that are not included in the panel of ENS but may be diagnosed in an early phase by enzymatic assessments. 

The pharmacologic treatment is based on nitrogen-scavenging drugs, such as sodium benzoate, sodium phenylbutyrate (NaPB), and glycerol phenylbutyrate (GPB) (Table 2) [6].

Sodium benzoate was first investigated for the alternative activation of pathways for nitrogen excretion in the 1980s [26], providing a new perspective for treating UCDs. It is unlicensed, has a high volume of administration and has an unpleasant taste [27].

NaPB was introduced in the mid-1990s [28,29]. It is still frequently used, being indicated as adjunctive therapy in the chronic management of UCDs, involving deficiencies of carbamylphosphate synthetase, ornithine transcarbamylase or argininosuccinate synthetase. It is indicated for the treatment of all patients with neonatal-onset presentation and those with late-onset disease who have a history of hyperammonemic encephalopathy [29]. It is very effective in lowering ammonia, but the regimen is challenging because of the poor palatability, body odor, large volume, high salt content and high frequency of administration [30].

GPB has been available since 2016–2018 [31]. It is currently approved for use in the USA and Europe for patients of all ages with UCDs who are insensitive to protein restriction diet and/or amino acid supplementation alone. GPB consists of a tasteless, odorless and is administered in a small volume, resulting in increased ease of the therapeutic regimen [31,32]. Experiences in switching patients from either sodium benzoate or NaPB to GPB have been reported to maintain metabolic balance and improve the quality of life of patients and caregivers [27,30,33,34]. Overall, the evolution of nitrogen scavengers progressively improved the management of UCDs, increasing adherence to treatment and quality of life, thus indirectly resulting in improved outcomes. On the basis of their clinical experience with nitrogen scavengers, the authors are in line with the published studies [27,30,33,34] supporting the preferential use of a scavenger with favorable patient response and proven pharmacological efficacy.

Symptomatic patients with suspected distal UCDs before NBS results should be treated immediately [6]. The first interventions include stopping protein intake, starting IV 10% glucose with electrolytes (Na^+^, K^+^), initiation of ammonia scavengers (L-arginine, sodium benzoate, glycerol phenylbutyrate) and collection for plasma amino acids and urinary orotic acid without postponing initiation of treatment. The choice of the drugs is based on amino acid value: sodium benzoate reduces glycine, and phenylbutyrate decreases glutamine. Patients with a hyperammonemia crisis should be immediately referred to a specialist center.

On the other hand, mitochondrial UCDs (*CPS1*, *OTC* and *NAGS1*), which are not currently included in the Italian ENS panel but are present in the Newborn Screening ACT Sheets and Algorithms, seem to be eligible for a strict follow-up and early treatment [35].

OTC-affected males are at risk of unpredictable hyperammonemia and must be treated with ammonia scavengers (L-arginine, sodium benzoate, glycerol phenylbutyrate) and low-protein diet [24]. On the contrary, asymptomatic females with early-onset OTC in the family should have no therapy. They could eventually start ammonia scavengers if the amino acids profile is altered or in case of stressful conditions, such as delivery or major surgery [24].

To reduce the risk of unnecessary drug usage and treatment of children with positive ENS, variants analysis [32,36] and, if available, enzymatic prediction by biallelic expression system (ASS1; ASL) [37] should be used to predict the clinical phenotype.

For asymptomatic patients with a positive family history for early onset UCD, measures for minimizing the stress of delivery should be considered: delivery in a hospital with a specialized metabolic unit; transfer the newborn to the neonatal unit; central venous catheter positioning; start IV glucose 10% (4 mL/kg/h; 6–8 mg/kg/min) with appropriate electrolytes (Na^+^, K^+^) within 30 min and initiate protein-free feed/infant formula; start every 6 h 50 mg/kg of both sodium benzoate and L-arginine. During the first hours of life, the measure of plasma ammonia at 6 h and if <80 μmol/L again every 6 h and if ammonia reaches 80–150 μmol/L, re-assay in 4 h; while, if ammonia > 150 μmol/L or if the baby becomes unwell repeat ammonia assay immediately, stop feeds and follow UDCs guidelines; amino acids should be assessed at 12 h of life [6].

Interventions at birth for asymptomatic patients with a positive family history of late-onset UCDs include: start glucose infusion only if the birth was complicated (birth asphyxia, etc.); give first-stage infant formula ≤ 6 g protein/day (corresponding to 130 mL infant formula/kg/d for a 3.5 kg baby and then increase over a few days to 150 mL/kg/day); breastfeeding on demand is possible, but top-up feeds are required to compensate for low volume and low energy on days 1 and 2. In asymptomatic patients, plasma ammonia and amino acids can be assessed at 24 h of age; otherwise, in case of symptoms, follow the current UCD guidelines [6].

The last statement of this section advises that “In the chronic management of symptomatic patients with positive NBS outside the acute episode, arginine supplementation and nitrogen scavengers are recommended”. Overall, this counsel agrees with Häberle’s proposed guidelines, which avoid drug recommendations [6]. Table 2 presents the characteristics of the ammonia scavengers used in the clinical practice for UCDs.

Patients diagnosed with distal UCDs by ENS should be monitored in the long-term follow-up for growth, head circumference, hair loss, skin rashes, vitamin deficiency, neurological and neurocognitive assessment at regular intervals depending on clinical situation, liver size and structure by ultrasound and dietary assessments. Laboratory monitoring consists of detecting ammonia (80 µmol/L as upper limit), plasma amino acids (glutamine not exceeding 1000 µmol/L) 3–4 h after the last meal at the same time, blood benzoate or phenylbutyrate assays if available, orotic acid in urine. Additionally, neuroimaging should be carried out (magnetic resonance spectroscopy, diffusion tensor imaging) every 2 years (if anesthesia is not required), even without neurological and cognitive impairment. The timing of follow-up should be decided by case evaluation, based on IQ measure (which is also necessary for milder diseases), monitoring of psychosocial factors, and quality of life. 

In the authors’ experience, different approaches are used according to the level of enzymatic deficiency. Patients affected by mild citrullinemia maintain breastfeeding.

Follow-up, diet and therapy are tailored based on biochemical values (glutamine, arginine, ammonia levels, orotic acid, etc.), clinical history (neurocognitive assay and psychomotor assessment) and familial history (the history of the affected siblings). 

Also, the authors suggest that patients should be clinically evaluated monthly when recalled for a positive ENS, then every 2–3 months to the end of the first year and then every 3–6 months or 6–12 months in the second year, depending on severity. It seems to be strictly necessary to monitor the clinical evolution of the disease and also to evaluate the patient’s tolerance and severity of the disorder. Clinical and biochemical data obtained in such controlled time points can personalize the treatment. Special attention is given to protein tolerance during weaning, and blood exams are checked more frequently during pregnancy [38].

**Table 2:** Pharmacologic profile of available ammonia scavengers, according to European data sheets. The recommended doses reported here may differ from protocols used by the authors.

	Glycerol Phenylbutyrate	Sodium Phenylbutyrate	Sodium Benzoate/Sodium Phenylbutyrate
**Registered**	Yes	Yes	No
**Indication**	Deficiencies of CPS1, OTC, ASS, ASL, ARG and HHH	Deficiencies of CPS1, OTC or ASS	Not available
**Age**	Children and adults, any age	Children and adults, any age	Not available
**Pharmaceutical form**	Oral liquid 1.1 g/mL	Tablets, granules	Many presentations
**Daily dose**	4.5 mL/m^2^/day to 11.2 mL/m^2^/day	450–600 mg/kg/day in children weighing < 20 kg 9.9–13.0 g/m^2^/day in children weighing > 20 kg, adolescents and adults	Not available
**Method of administration**	With meals directly into the mouth Possible use of naso-gastric tube	With meals, mix with liquids Possible use of naso-gastric tube	
**Sodium content**	None	62 mg/tablet 124 mg/g	Yes, variable with different presentations
**Clinical efficacy**	**Study 1** (44 adults, controlled, non-inferiority, cross-over, 4 weeks): vs. sodium phenylbutyrate. Results: non-inferior for 24 h AUC for ammonia **Study 2** (51 adults, uncontrolled, open, 12 months). Results: Mean fasting venous ammonia values were normal **Study 3 and 4** (pediatric patient, open, switch from sodium phenylbutyrate, 14 and 10 days). Results: non-inferior for ammonia control **Long-term pediatric studies** (49 pediatric patients, part of study 1 and extension of studies 3 and 4, 12 months). Results: mean fasting venous ammonia within limits **Study 5** (45 pediatric patients, open, up to 5.9 years). Results: mean fasting venous ammonia within limits at 24 months **Study under 2 months age** (16 patients, uncontrolled, open, 24 months, naïve patients or switch from sodium phenylbutyrate). Results: successful transition in 3 days, mean normalized venous ammonia values were normal in the long term **Study 2 months to 2 years** (10 patients, uncontrolled, open, 24 months). Results: nine successful transitions in 4 days	See studies vs. glycerol phenylbutyrate	Not available
**Elimination**	Metabolized by pancreatic enzymes; metabolites are eliminated in the urine; 80–100% of the medicinal product is excreted by the kidneys within 24 h as phenylacetylglutamine	Metabolized to phenylacetate that forms phenylacetylglutamine in liver and kidney; 80–100% of the medicinal product is excreted by the kidneys within 24 h as phenylacetylglutamine	Converted to benzoic acid. The individual maximum rate of metabolism can be estimated from the urinary excretion rate of hippuric acid 1.5 to 3 h after the single oral dose

CPS: carbamoyl phosphate synthetase, OTC: ornithine carbamoyltransferase, ASS: argininosuccinate synthetase, ASL: argininosuccinate lyase, ARG: arginase I, HHH: ornithine translocase deficiency hyperornithinemia–hyperammonemia–homocitrullinuria syndrome. Data taken from the Ravicti SmPC [31], and the Pherurane SmPC [29].

## 4. Discussion and Conclusions

This article provides detailed counsel for the management of mild UCDs encompassing the role of ENS, biochemical and genetic variant analyses in establishing and completing distal UCD diagnosis. Moreover, counsels for clinical surveillance during the first year, treatment choice and criteria evaluation for long-term follow-up are also provided here. Such counsels sprang from the shared experience of an expert group of specialists in the field and the reappraisal of the current literature. 

At present, knowledge on the natural history, the treatment and clinical outcome of individuals with mild UCDs is scant; The biochemical parameters available by NBS may allow to decide therapy initiation on the basis of glutamine, citrulline and arginine levels. The following investigation including a genetic profile will allow to identify the phenotype and drive the decision to start dietetic or pharmacologic therapy. Altered values of one of these amino acids and/or the presence of a genetic profile predictive of severe phenotype should prompt the decision to start dietetic or pharmacological therapy [39]. The American Urea Cycle Disorders Consortium [40] and the European registry and network for Intoxication type Metabolic Diseases [41] aim to improve the knowledge of the natural history, treatment and clinical outcome of individuals with mild UCDs [24]. Both consortia focus on elucidating key physiopathology, early prediction of clinical severity, evaluating safety and efficacy of dietary treatment, pharmacological therapy, and liver transplantation, developing evidence-based consensus care recommendations, and empowering patients and their families [21].

These objectives will be attained in several years and the present situation, the shared opinion of a group of experts can help clinicians in their practice and in developing future perspectives. The authors reap the benefits of the traditional use of ENS in Italy, which demonstrated that UCDs cannot be considered only in their acute forms and that late-onset cases can be detected in a relevant proportion of patients if suitable diagnostic processes are available. The relevance of ENS is confirmed by the recently published analysis of the two large databases gathered by the American and the European consortia [21]. Therefore, strategies to implement ENS with clinical observation and enzyme assessment to predict the patient’s evolution is of main importance [21].

The authors must acknowledge that in the absence of clinical trials, the treatment of mild UCDs is currently based on the protocols for acute cases, with limited changes. On the other hand, they are confident that early diagnosis of late-onset cases may prevent poor outcomes, confirming previous observations [42,43].

According to the authors, this is the first set of counsels focused on the management of mild UCDs produced by a group of experts and intended to be a guide for clinical practice. These counsels can provide a useful framework for managing mild UCDs and a basis for the design of focused clinical studies, until further evidence is generated in the future. 

The strength of this article is that statements were produced to achieve an overall consensus on the management of mild UCDs, from their identification by ENS to the follow-up of patients. Additionally, the advisors are a restricted group with high-level and extensive experience with ENS and with managing metabolic disorders. Finally, although produced by a national group, these counsels may be the basis to draw protocols to be implemented in centers worldwide. 

Nevertheless, some limitations of this study should be acknowledged. All experts were recruited from leading hospitals and ENS centers in Italy, which selected a limited number of panelists and resulted in a selected information gathered from centers of excellence and not from smaller hospitals. Despite this, the expertise of panelists was granted by the substantial experience in ENS in the Italian healthcare system. 

In conclusion, this project represents a preliminary step for developing guidelines or more advanced advice by a wider panel. Once further evidence is gathered, it will be possible to produce updated guidance on the management of mild UCDs. 

## Figures and Tables

**Table 1:** Final items, statements and degree of consensus.

Final Items	Final Statements	Score (%)					Degree of Consensus (%)
		Score 1	Score 2	Score 3	Score 4	Score 5	
The role of expanded newborn screening in UCDs	NBS for distal urea cycle disorders (high citrulline, high arginine, high argininosuccinic acid) is effective both in terms of sensitivity (low false-negative rate) and specificity (low false-positive rate)	0	0	0	12.5	87.5	100
	NBS for proximal UCDs (low citrulline, high orotic acid, high glutamine) is less efficient regarding specificity and sensitivity	0	0	0	50	50	100
	When neonatal screening yields positive results for UCDs, managing the patient involves considering clinical and metabolic parameters, family history, and molecular data	0	0	0	0	100	100
	If the citrulline levels exceed 100 µmol/L, it is crucial to recall the patient immediately	0	0	12.5	25	62.5	87.5
Clinical genotype/phenotype correlation	Under an emergency regime, a biochemical confirmation test based on plasma amino acid assessment is mandatory for the diagnosis and specific treatment	0	0	0	25	75	100
	Genetic testing is mandatory for confirmation	0	0	0	0	100	100
	If no mutations are found in the genes involved in the screened pathologies, a second-line genetic screening must be performed, investigating genes associated with secondary hyperammonemia	0	12.5	0	12.5	75	87.5
	It is always important to report all variants, even if a person shows no signs or symptoms of the disease	0	0	12.5	12.5	75	87.5
	Prediction of enzymatic defect for severe form, assessed by biallelic expression system and positive family history, should guide the pharmacological treatment in the pre-symptomatic period	0	0	0	37.5	62.5	100
Diet therapy	Regular nutritional counseling must be offered for dietetic evaluation, growth assessment, and family weaning instruction in the first year of life	0	0	0	0	100	100
	During catabolic events (i.e., gastroenteritis, fever, starvation) or protein overload, patients should be treated with an integrated approach of aetiologic and supportive therapy and a low-protein diet (≤1 g/kg/day) with arginine supplementation	0	0	0	25	75	100
	When dealing with mild UCDs, especially in late-onset forms diagnosed through NBS, the switch from a “wait and see” approach to an active therapeutic approach should be based on the abnormality of biochemical data and/or genotype profile	0	0	0	25	75	100
	The decision about which treatment to initiate, between diet and pharmacological treatment, should consider family social/behavioral capabilities. Breastfeeding can be maintained even after initiating the treatment unless there is an increase in ammonemia	0	0	0	12.5	87.5	100
	Patients with predictive severe genotypes should be treated with a combined diet and pharmacological approach	0	0	0	0	100	100
	For patients with non-severe predictive genotypes, conducting anamnestic and ammonia evaluations during follow-up is important. In addition, they should receive an emergency scheme and a case-by-case evaluation for preventive combined treatment	0	0	0	0	100	100
Pharmacological treatment	In asymptomatic patients with positive NBS, ammonia and plasma amino acid levels should guide pharmacological treatment	0	0	0	12.5	87.5	100
	If neonatal screening confirms elevated levels of argininosuccinic acid and either normal or mildly increased levels of citrulline at plasma amino acid (>100 μmol/L), it is recommended to start oral arginine supplementation	0	0	12.5	0	87.5	87.5
	In the chronic management of symptomatic patients with positive NBS outside the acute episode, arginine supplementation and nitrogen scavengers are recommended	0	0	0	0	100	100
Follow-up	During the first year, the follow-up has a diagnostic purpose and should be as strict as possible in order to know the patient, the disease, and its variables	0	0	0	25	75	100
	A strict follow-up allows for prescribing a tailor-made therapy based on clinical and biochemical data to avoid the risk of overtreatment	0	0	0	12.5	87.5	100
	Regular follow-up with outpatient pediatric visits should monitor anthropometric parameters, biochemical markers (ammonia, liver function, plasma amino acids, orotic acid), nutritional state (protein intake), and neurological/psychomotor examination	0	0	0	12.5	87.5	100
	The follow-up programs should be modified according to the personal needs of patients, taking into consideration acute episode frequency, growth parameters, nutrition, diet adherence, routine blood tests, and neurocognitive development	0	0	12.5	12.5	75	87.5
	During the pre-symptomatic period, predicting potential enzymatic defects and studying the siblings is essential; this information may guide follow-up care	0	0	0	50	50	100
